# Hsa_circ_0005397 promotes hepatocellular carcinoma progression through EIF4A3

**DOI:** 10.1186/s12885-024-11984-6

**Published:** 2024-02-21

**Authors:** Liu-Xia Yuan, Mei Luo, Ruo-Yu Liu, Hui-Xuan Wang, Lin-Ling Ju, Feng Wang, Ya-Li Cao, Zhong-Cheng Wang, Lin Chen

**Affiliations:** 1 Institute of Liver Diseases, Nantong Third People’s Hospital, Affiliated Nantong Hospital 3 of Nantong University, 226000 Nantong, Jiangsu China; 2 Nantong Third People’s Hospital, Medical School of Nantong University, 226000 Nantong, Jiangsu China; 3 Medical School of Nantong University, Affiliated Hospital of Nantong University, 226000 Nantong, Jiangsu China; 4 Preventive Health Department, Nantong Third People’s Hospital, Affiliated Nantong Hospital 3 of Nantong University, 226000 Nantong, Jiangsu China; 5 Hepatology Department of integrated Chinese and Western Medicine, Nantong Third People’s Hospital, Affiliated Nantong Hospital 3 of Nantong University, 226000 Nantong, Jiangsu China

**Keywords:** Hepatocellular carcinoma, hsa_circ_0005397, EIF4A3

## Abstract

**Purpose:**

The purpose of this study was to explore the expression and potential mechanism of hsa_circ_0005397 in hepatocellular carcinoma progression.

**Methods:**

Quantitative reverse transcription-polymerase chain reaction(qRT-PCR) was used to measure the expression level of hsa_circ_0005397 and EIF4A3 from paired HCC tissues and cell lines. Western Blot (WB) and immunohistochemistry (IHC) were used to verify the protein level of EIF4A3. The specificity of primers was confirmed by agarose gel electrophoresis. Receiver Operating Characteristic (ROC) Curve was drawn to analyze diagnostic value. Actinomycin D and nuclear and cytoplasmic extraction assays were utilized to evaluate the characteristics of hsa_circ_0005397. Cell Counting kit-8 (CCK-8) and colony formation assays were performed to detect cell proliferation. Flow cytometry analysis was used to detect the cell cycle. Transwell assay was performed to determine migration and invasion ability. RNA-binding proteins (RBPs) of hsa_circ_0005397 in HCC were explored using bioinformatics websites. The relationship between hsa_circ_0005397 and Eukaryotic Translation Initiation Factor 4A3 (EIF4A3) was verified by RNA Binding Protein Immunoprecipitation (RIP) assays, correlation and rescue experiments.

**Results:**

In this study, hsa_circ_0005397 was found to be significantly upregulated in HCC, and the good diagnostic sensitivity and specificity shown a potential diagnostic capability. Upregulated expression of hsa_circ_0005397 was significantly related to tumor size and stage. Hsa_circ_0005397 was circular structure which more stable than liner mRNA, and mostly distributed in the cytoplasm. Upregulation of hsa_circ_0005397 generally resulted in stronger proliferative ability, clonality, and metastatic potency of HCC cells; its downregulation yielded the opposite results. EIF4A3 is an RNA-binding protein of hsa_circ_0005397, which overexpressed in paired HCC tissues and cell lines. In addition, expression of hsa_circ_0005397 decreased equally when EIF4A3 was depleted. RIP assays and correlation assay estimated that EIF4A3 could interacted with hsa_circ_0005397. Knockdown of EIF4A3 could reverse hsa_circ_0005397 function in HCC progression.

**Conclusions:**

Hsa_circ_0005397 promotes progression of hepatocellular carcinoma through EIF4A3. These research findings may provide novel clinical value for hepatocellular carcinoma.

**Supplementary Information:**

The online version contains supplementary material available at 10.1186/s12885-024-11984-6.

## Introduction

Hepatocellular carcinoma (HCC) is the most common type of primary liver cancer worldwide [1]. Viral hepatitis, alcohol abuse, and nonalcoholic hepatic steatosis are the main risk factors for HCC [2, 3]. Because of the lack of early symptoms and signs, most HCC patients are already in the advanced stage at the time of diagnosis. Despite recent achievements in HCC treatment, its diagnosis and prognosis remain challenging [4, 5]. Therefore, seeking effective diagnosis and treatment is crucial. As reported in our previous study, plasma hsa_circ_0005397 represents a good value for HCC [6]. However, the underlying mechanisms of HCCprogression or metastasis have not been fully elucidated.

Circular RNAs (circRNAs) are noncoding RNAs that are formed through backsplicing of linear RNA, resulting in a circular structure [7]. Due to their stable structure, they are resistant to degradation [8]. Furthermore, circRNAs are widely distributed in body fluids and blood, underscoring their potential as stable biomarkers [9, 10]. Moreover, circRNAs participate in cancer pathogenesis, invasion and metastasis. For example, circ_103809 targets miR-620 to suppress proliferation and invasion in HCC [11], and hsa_circ_104348 modulates the miR-187-3p/RTKN2 axis in hepatocellular carcinoma progression [12]. Overall, reports indicate that circRNAs are promising biological targets for HCC diagnosis and prognosis.

Recently, knowledge of RBPs has increased substantially owing to the development of new technologies [13]. RBPs influence RNA biology post-transcriptionally, and an increasing amount of evidence suggests that abnormal RBPs expression and function are associated with cancer metastasis [14]. As reported, circRNAs regulate the biological activity of downstream targets by binding to RBPs [15]. EIF4A3 is a commonly studied RBP involved in posttranscriptional regulation [16, 17]. For example, EIF4A3 interacts with circ_0084615, promoting progression of colorectal cancer via miR-599/ONECUT2 [18]. Additionally, EIF4A3 binds to circ_0004296, inhibiting metastasis of prostate cancer [19]. Moreover, hsa_circ_0068631 recruits EIF4A3, which increases expression of c-Myc in breast cancer, showing an important role in cancer metastasis [20]. As reported, hsa_circ_0005397 is a kind of circRNA originating from RHOT1 that promotes HCC by regulating the miR-326/PDK2 axis [21]. Hsa_circ_0005397 might also be involved in other pathogeneses with various binding sites. In this study, we found a meaningful interaction between hsa_circ_0005397 and EIF4A3 in HCC progression.

## Materials and methods

### Clinical tissue samples

We collected 57 tumor tissues and paired adjacent tissues from HCC patients at Nantong Third People’s Hospital. Patients who received surgical treatment without any other treatment were chosen for the study. Tissues were immediately stored at -80 °C. The clinical data of all patients were collected. This study was approved by the Ethics Committee of Nantong Third Affiliated Hospital of Nantong University and were conducted in accordance with the Declaration of Helsinki. All participants signed informed consent forms in this study.

### Cell culture

The normal hepatocyte cell line (LO2) and HCC cell lines (SMMC-7721, SK-Hep1, HCCLM3, BEL-7404, Huh7) were acquired from Type Culture Collection Cell Committee of the Chinese Academy of Sciences (Shanghai, China). The cells were cultured in suitable medium containing 10% fetal bovine serum (Lonsera, USA) at 37 °C with 5% CO2.

### Construction of plasmids and siRNAs

SiRNAs targeting the negative control (si-NC), and hsa_circ_0005397 (si1, si2) were obtained from GenePharma (Suzhou, China). ShRNAs targeting the EIF4A3 (sh EIF4A3) and negative control (sh NC) were obtained from GenePharma (Suzhou, China). Overexpression plasmids, human hsa_circ_0005397 cDNA was synthesized and cloned into the pcDNA3.1 vector (pcDNA) and empty vector was used as the negative control (vector) which obtained from Shanghai (Gechem, China). We used lipofectamine 3000 and P3000 (Invitrogen, USA) for plasmid overexpression and lipofectamine 3000 for siRNA transfection according to the manufacturer’s instructions.

### RNA preparation and qRT-PCR

Total RNA was isolated by TRIzol reagent (Invitrogen, USA) and the concentration of total RNA was detected by NanoDrop spectrophotometer (Thermo Fisher Scientific, USA). CDNA was synthesized by Synthesis Kit (Thermo Fisher Scientific, USA), and SYBGreen I Master Mix (Roche, Germany) was used for qRT-PCR. 18 S was used as an endogenous control. All data were normalized to internal control, and relative expression was calculated by the 2–ΔΔCT method. Hsa_circ_0005397 primers were purchased from Guangzhou (Geneseed, China), and other primers were purchased from Shanghai (Sangon Biotech, China). The primers used in this study were as follows: hsa_circ_0005397 (Forward: 5′-GACAAAGACAGCA GGTTCCT-3′; Reverse: 5′-CTC​TGT​TCT​GCT​TCT​GAG​TA-3′); EIF4A3 (Forward: 5’-CAGGGCGTGTTTTTGATATGAT-3’; Reverse: 5’-ATCAGCTTCATCCAAAACCAAC-3’; RHOT1 (Forward: 5’-CTGATTTCTGCAGGAAACACAA-3’; Reverse: 5’-GCAAAAACAGTAGCACCAAAAC-3’); and 18 S rRNA (Forward: 5’-GTAACCCGTT GAACCCCATT-3’; Reverse: 5’-CCATCCAATCGGTAGTAGCG-3). After qRT-PCR, PCR products were examined by 2% agarose gel electrophoresis. The experiments were performed at least three times independently with triplicate samples.

### RNase R resistance assay

For RNase R treatment, 1 µg of total RNA was incubated with or without 0.1 µL RNase R (20 U/µL) and 1µL 10X Reaction Buffer for 0.5 h at 37 °C. After purified, qRT-PCR was used to examine the levels of hsa_circ_0005397 and RHOT1.

### Western blotting

Proteins were extracted in RIPA Lysis buffer (Beyotime, China) with protease and phosphatase inhibitors. Protein lysates were separated by 10% SDS-PAGE gels and transferred to PVDF membrane (Millipore Corporation, China). After blocking in non-fat milk, membranes were incubated with primary antibody overnight at 4 ℃, and incubated with a secondary antibody for 1 h at room temperature. The primary antibody used as follows: anti-EIF4A3 (1:1000, Proteintech, China), anti-GAPDH (1:20000, Proteintech, China), HRP-conjugated goat anti-rabbit IgG (1:1000, Beyotime, China). The bands were visualized by an enhanced chemiluminescence detection system (Tanon, China).

### Immunohistochemistry (IHC) examination

After dewaxed with xylene and gradient of ethanol concentration. The slices were subjected to citrate microwave heating treatment for antigen retrieval. 3% H2O2 was used to block endogenous peroxidase activity. Next, the slices were incubated with anti-EIF4A3 (1:100, Proteintech, China) overnight at 4℃ and subsequently with horseradish peroxidase-conjugated anti-mouse/rabbit secondary antibody (1:1000, Changdao Biotechnology, China) for 1 h at room temperature. After washed, the slices were stained with hematoxylin and 3,3’-diaminobenzidine (DAB, MaixinBio, China). Finally, the slices were imaged in five randomly fields. Finally, two pathologists evaluated all stained sections independently. The staining intensity was scored as 0 (no staining), 1 (weak staining), 2 (medium staining) and 3 (strong staining); the degree of staining was scored as 0 (≤ 10%), 1 (> 10-25%), 2 (> 25-50%), 3 (> 50-75%) or 4 (> 75%). Scoring was based on the product of the staining intensity score and degree score.

### Nuclear and cytoplasmic extraction

The nuclear and cytoplasmic fractions of RNA were collected with extraction kits (Thermo Fisher Scientific, USA) according to the manufacturer’s instructions. After collected, the purified RNA was extracted and detected by qRT-PCR.

### Actinomycin D assay

2.5 µg/mL Actinomycin D (Merck, Germany) was added to cells and cultured for 8 h. TRIzol reagent (Invitrogen, USA) was added at 0, 2, 4, 6, and 8 h to collect RNA. after collected, the purified RNA was extracted and detected by qRT-PCR.

### Cell counting kit-8 (CCK-8) assay

After 48 h, transfected cells were collected, and 3,000 cells/well were seeded in 96-well plates. After incubation with CCK-8 solution (MCE, USA) for 2 h, the OD value was measured at 450 nm by using a microplate reader (Thermo Fisher Scientific, USA).

### Colony-formation assay

After transfection, total of 3,000 cells were added to a six-well plate for two weeks and fixed with 4% formaldehyde (Beyotime, China). After stained with 0.1% crystal violet (Sigma, USA), cell clones were counted and analyzed under a light microscope.

### Transwell assay

After transfection for 48 h, 5 × 10^4^ cells were suspended in 200µL serum-free medium and seeded into the upper chamber (Costar, USA) with or without precoated with 40 µl Matrigel (BD Biosciences, USA), and 600µL MEM media containing 20% FBS was put into bottom of chambers. After incubated for 48 h, cells were fixed with 4% paraformaldehyde for 15 min, and stained with 0.1% crystal violet (Sigma, USA) for 30 min and photographed under a microscope (Olympus, Japan). Cell counts were counted in five randomly fields, The experiments were performed at least three times independently with triplicate samples.

### Website acquisition

We used The Cancer Genome Atlas Program ( https://portal.gdc.cancer.gov/ ) to obtain the expression profile of EIF4A3. CircInteractome (https://circinteractome.nia.nih.gov/) and CircFunBase (https://bis.zju.edu.cn/CircFunBase/index.php) were used to search RNA-binding proteins matched to hsa_circ_0005397. This platform contributes to circRNA-protein studies [22, 23].

### Cell cycle assay analysis

The DNA content test kit (Solarbio) was used to evaluate cell cycle. After transfection, the treated cells were collected and fixed in ice-cold 70% ethanol overnight. After removing the 70% ethanol, the cells were washed by PBS. Cells were resuspended with 100 µL of RNase A solution at 37 °C for 30 min, and incubated with 400 µL of PI staining solution for 30 min at 4 °C in the dark. Cell cycle was detected by flow cytometry (Bio-Rad, USA).

### RNA immunoprecipitation (RIP) assay

RIP Kit (Geneseed, China) was used to perform RIP assay. A total of 1 × 10^7^ cells were lysed, some of them was collected to acquire input RNA, while another was incubated with magnetic beads conjugated with human anti-EIF4A3 (anti-EIF4A3) or anti-immunoglobulin G (anti-IgG) antibody at 4 °C, after incubated, the purified RNA was extracted and detected by qRT-PCR.

### Statistical analysis

Data, based on at least three independent experiments, are presented as the mean ± standard deviation (SD) and were analyzed by GraphPad Prism 7.0 (GraphPad Software, USA). The t test and one-way analysis were employed to analyze differences between two groups or among more than two groups. Correlation was calculated by Pearson’s correlation analyses. *P* values < 0.05 were considered significant.

## Results

### Characteristics of hsa_circ_0005397 in hepatocellular carcinoma

**Fig. 1:**
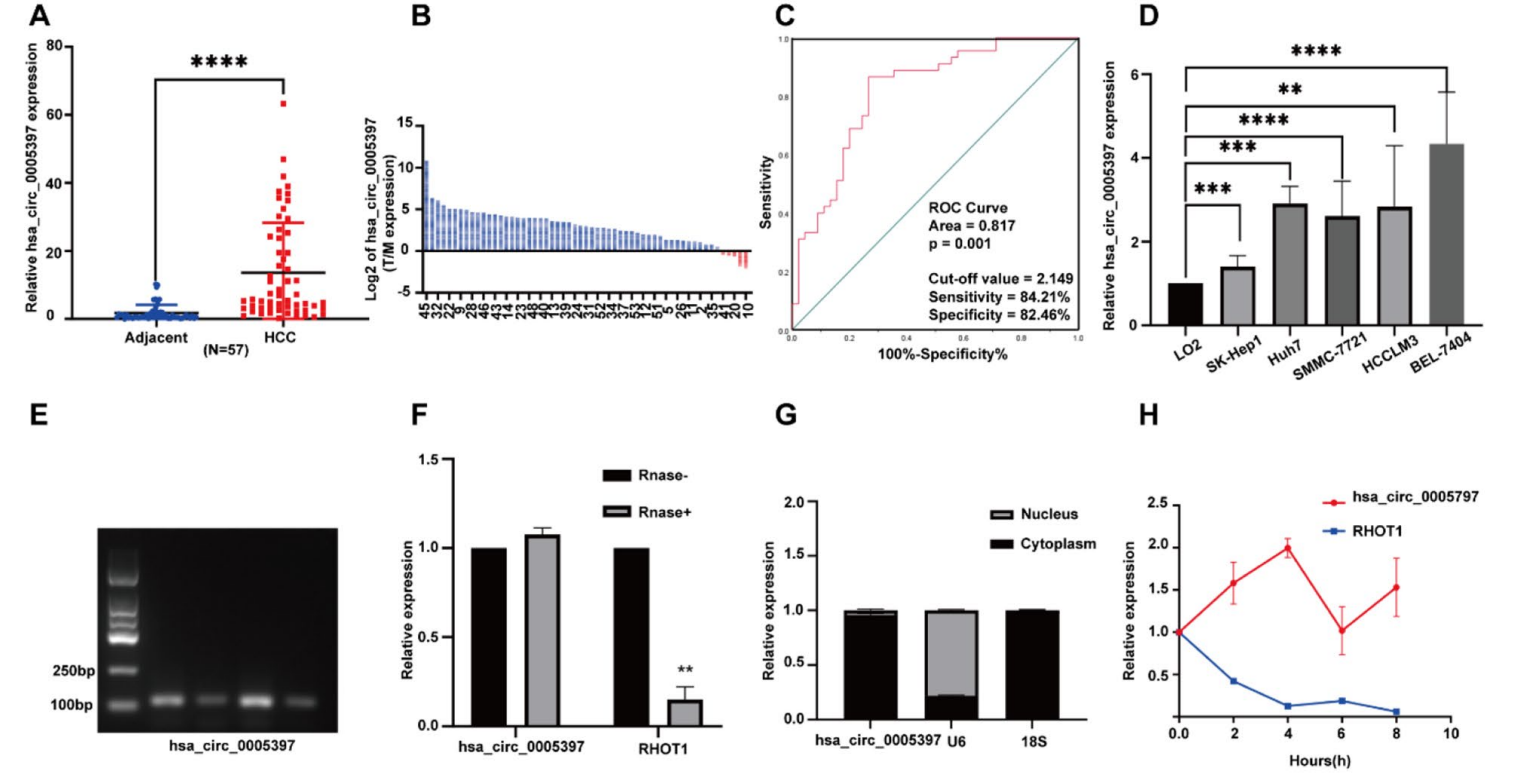
The characteristics of hsa_circ_0005397 in hepatocellular carcinoma. (**A**) qRT-PCR detected the expression level of hsa_circ_0005397 in HCC tissues (*n* = 57). (**B**) The relative expression level of hsa_circ_0005397 in HCC tissues were normalized to normal tissues (*n* = 57). (**C**) Construction of an ROC curve for HCC patients based on hsa_circ_0005397 expression. (**D**) qRT-PCR analyzed the expression of hsa_circ_0005397 in cell lines. (*P < 0.05, **p < 0.01, ***p < 0.001). (**E**) The PCR product of qRT-PCR was run on 2% agarose gel, showing a single electrophoresis band of has_circ_0005397. (**F**) qRT-PCR analyzed relative expression after treatment with or without RNase R in BEL-7404. (**G**) qRT-PCR was used to measure the expression level of hsa_circ_0005397 in the nuclear and cytoplasmic. (**H**) qRT-PCR was used to detected the relative expression of hsa_circ_0005397 and RHOT1 mRNA in BEL-7404 cells treated with Actinomycin D at different time points

Hsa_circ_0005397 was found to be upregulated in HCC tissues (Fig. 1A-B), and higher hsa_circ_0005397 was associated with TNM stage (*n* = 57, *p* = 0.018) and tumor size (*n* = 57, *p* = 0.025) (Table 1). According to ROC analysis, the area under the ROC curve was 0.8763 (95% CI 0.81–0.93), with a sensitivity of 84.21% (95% CI 0.72–0.91) and a specificity of 82.46% (95% CI 0.71–0.90), the ideal cut-off value was 2.149 (Fig. 1C). HCC cell lines presented higher expression compared with LO2 cells (Fig. 1D), and the primers were specific (Fig. 1E). We noted that hsa_circ_0005397 was derived from RHOT1, the results showed that RNase R could digest linear RHOT1 RNA but not hsa_circ_0005397, suggesting a cycle structure for hsa_circ_0005397 (Fig. 1F). Furthermore, the distribution of hsa_circ_0005397 was explored via cell fractionation, with hsa_circ_0005397 mostly located in the cytoplasm (Fig. 1G). To investigate the stability of hsa_circ_0005397, we conducted an actinomycin D experiment, results shown that the expression level of hsa_circ_0005397 was not significantly decreased, which confirmed that hsa_circ_0005397 was more stable to actinomycin D treatment than liner mRNA RHOT1 (Fig. 1H).

**Table 1:** Relationship between clinicopathological characteristics and hsa_circ_0005397 expression in patients

Clinicopathological characteristics		Hsa_circ_0005397 expression			*P* value
		Low expression	High expression		
Age(years)					0.881
> 60		12(42.9%)	13(44.8%)		
≤ 60		16(57.1%)	16(55.2%)		
Gender					0.612
Male		88821(75%)	20(69.0)		
Female		7(25%)	9(31%)		
Tumor size(cm)					0.025
<4.5	17(60.7%)		9(31%)		
≥ 4.5		11(39.3%)	20(69%)		0.190
Vascular invasion					
Yes		21(75.0%)	17(58.6%)		
No		7(25.0%)	12(41.4%)		
Tumor Stage					0.018
I-II		13(46.4%)	5(17.2%)		
III-IV		15(53.6%)	24(82.8%)		

According to TNM Classification by the American Joint Committee on Cancer (AJCC) /Union for International Cancer Control (AJCC/UICC). *P* value ≤ 0.05 indicated a significant difference

### Hsa_circ_0005397 affects proliferation in HCC cell lines

**Fig. 2:**
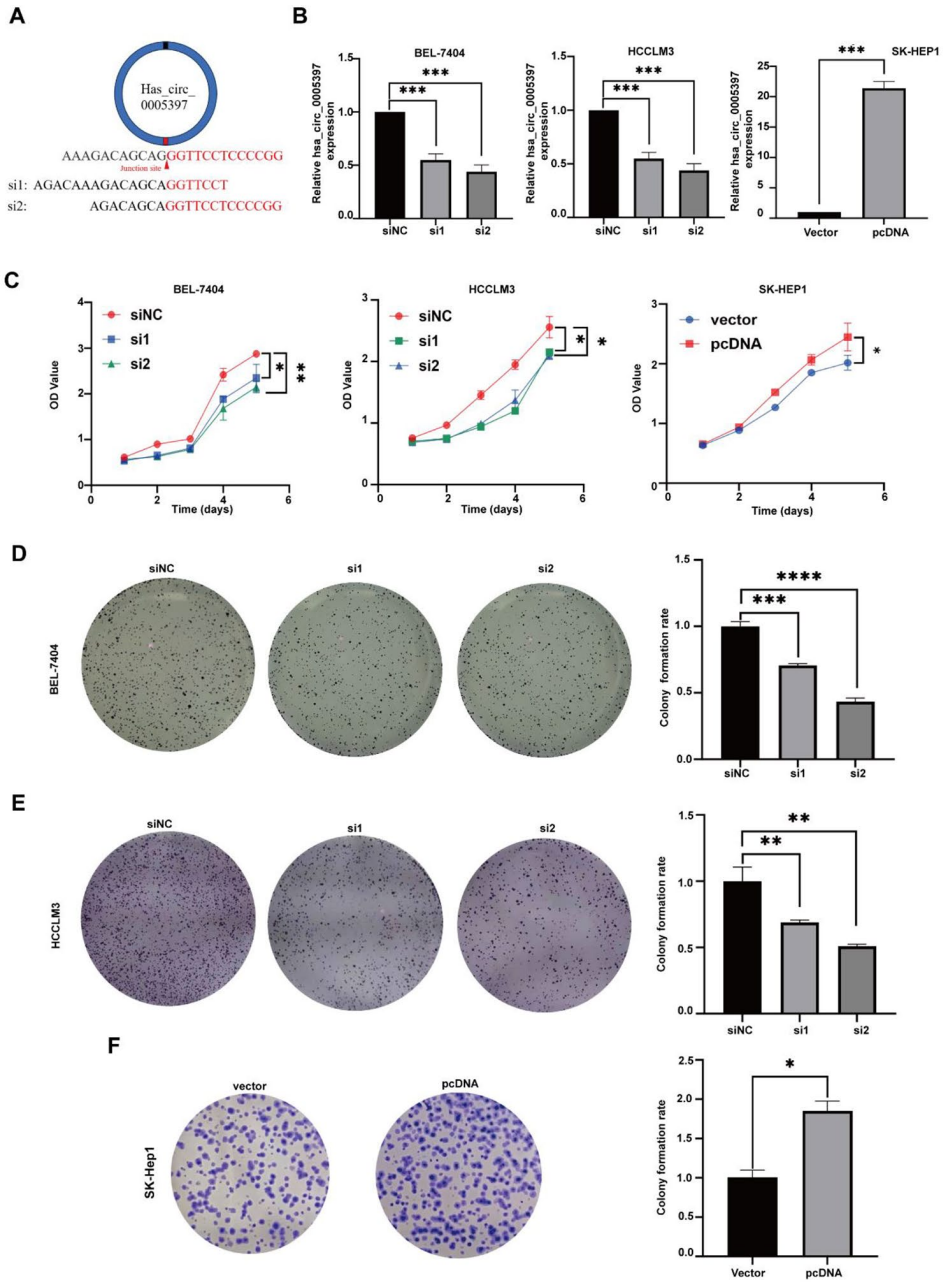
Hsa_circ_0005397 affects proliferation in HCC cell lines. (**A**) two siRNAs target the back splicing junction of hsa_circ_0005397. (**B**) qRT-PCR detected the expression of hsa_circ_0005397 in BEL-7404, HCCLM3 and SK-Hep1 cells. (**C**) Cell proliferation was determined by CCK-8 assays in BEL-7404, HCCLM3 and SK-Hep1 cells. (**D**) Cell proliferation ability was assessed by Colony formation assays after knocking down of hsa_circ_0005397 in BEL-7404 cells. (**E**) Cell proliferation detection was measured by Colony formation assays after knocking down of hsa_circ_0005397 in HCCLM3 cells. (**F**) Cell proliferation ability was assessed by Colony formation assays after overexpression of hsa_circ_0005397 in SK-Hep1 cells. Colony formation rate was analyzed by colony counts. *P < 0.05, **p < 0.01, ***p < 0.001

To explore the functions of has_circ_0005397 in HCC cells, two siRNAs targeting the back splicing region of has_circ_0005397 were transfected into BEL-7404 and HCCLM3 (Fig. 2A). What’s more, we constructed the hsa_circ_0005397 overexpression vector pcDNA3.1- hsa_circ_005397 (pcDNA) and control vector (vector) into SK-Hep1. qRT-PCR analysis revealed that si-hsa_circ_0005397 (si1, si2) significantly silenced expression of hsa_circ_0005397 in HCCLM3 and BEL-7404 cells but that high-efficiency overexpression was observed in SK-Hep1 cells (Fig. 2B). CCK-8 assays showed that overexpression of hsa_circ_0005397 promotes cell proliferation; consistently, silencing hsa_circ_0005397 inhibited cell proliferation (Fig. 2C). Compared with the negative control, the hsa_circ_0005397-overexpression group showed an increased colony counts in SK-Hep1 cells, in contrast to the hsa_circ_0005397-depletion group (Fig. 2D-F).

### Hsa_circ_0005397 affects migration and invasion capacity in HCC cell lines

**Fig. 3:**
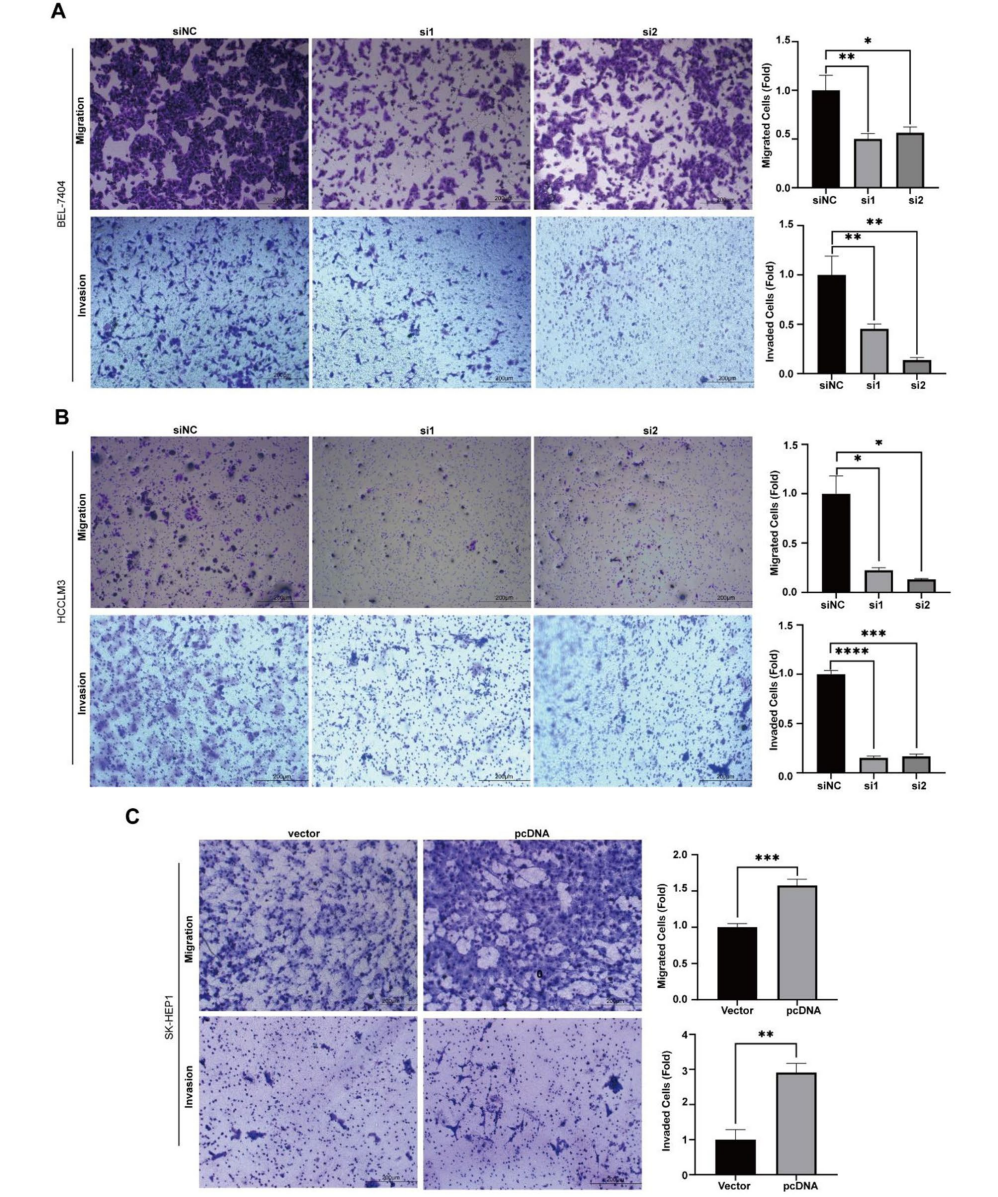
Hsa_circ_0005397 affects migration and invasion in HCC cells. (**A**) Transwell assays were used to detect cell migration and invasion of BEL-7404 cells transfected with siNC, si1and si2 (Magnification, 20X; Scale bars, 200 μm). (**B**) Cell migration and invasion abilities were determined by transwell assays in HCCLM3 cells transfected with siNC, si1 and si2 (Magnification, 20X; Scale bars, 200 μm). (**C**) The migratory and invasive capabilities of hsa_circ_0005397-overexpression SK-Hep1 cells (Magnification, 20X; Scale bars, 200 μm). *P < 0.05, **p < 0.01, ***p < 0.001

Furthermore, we explored the effects of hsa_circ_0005397 on migration and invasion in HCC cell lines by transwell assays. Relative migration and invasion rate were increased in the hsa_circ_0005397-overexpression group in SK-Hep1 cells (Fig. 3C), but decreased in the hsa_circ_0005397-depletion group in BEL-7404 and HCCLM3 cells, with significant differences (Fig. 3A-B).

### Hsa_circ_0005397 affects the cell cycle in HCC cell lines

**Fig. 4:**
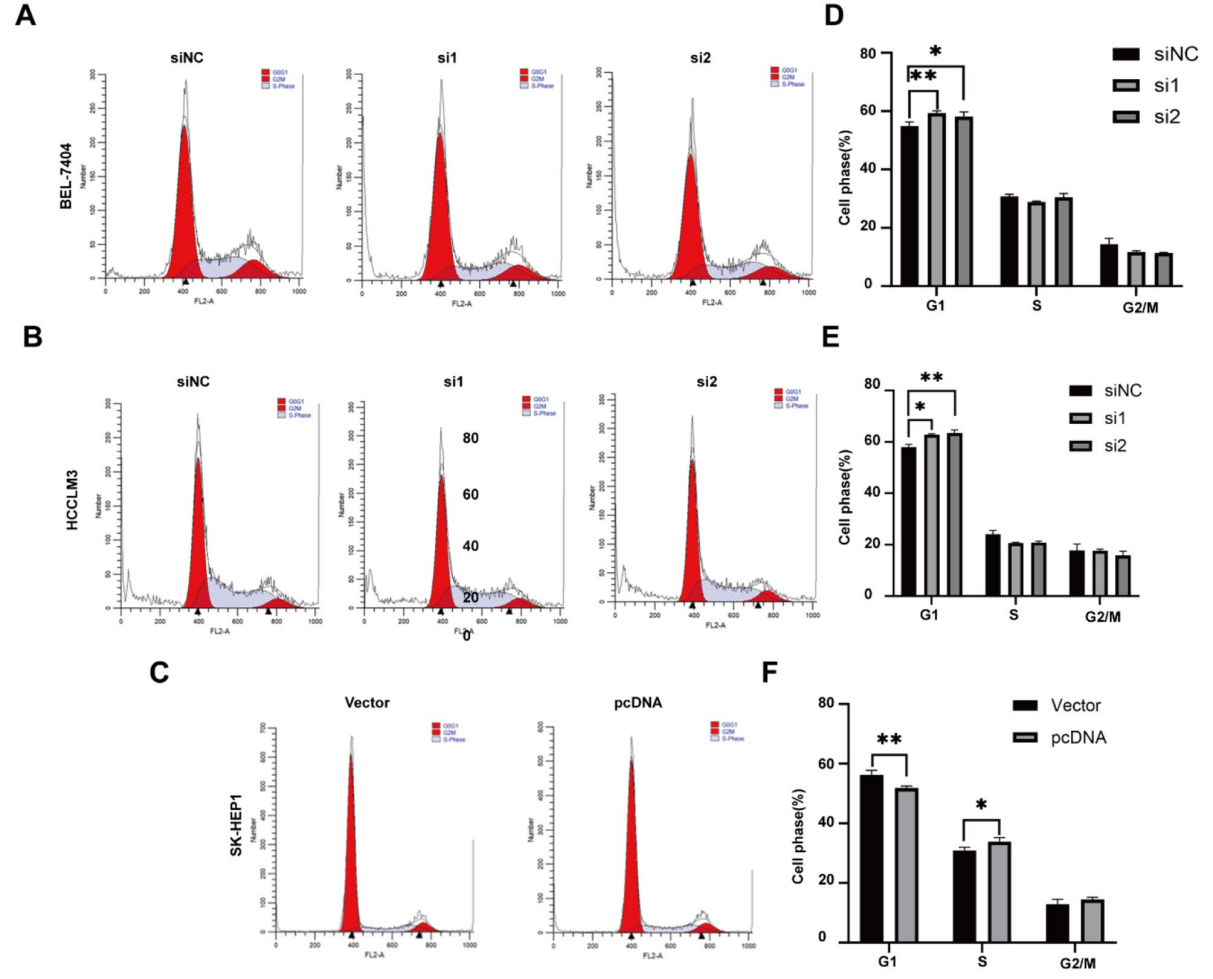
Hsa_circ_0005397 affects the cell cycle in HCC cells. (**A**) The FACS analysis of the cell cycle distribution was shown in BEL-7404 cells transfected with siNC, si1and si2. (**B**) The FACS analysis of the cell cycle distribution was shown in HCCLM3 cells transfected with siNC, si1and si2. (**C**) The FACS analysis of the cell cycle distribution was shown for has_circ0005397-overexpression SK-Hep1 cells. (**D**) Comparison of the cell cycle distribution of has_circ0005397-knockdown BEL-7404 cells. (**E**) Comparison of the cell cycle distribution of has_circ0005397-knockdown HCCLM3 cells. (**F**) Comparison of the cell cycle distribution of has_circ0005397-overexpression SK-Hep1 cells. *P < 0.05, **p < 0.01, ***p < 0.001

Furthermore, the cell cycle was evaluated, and the results indicated that the percentage of cells in G0/G1 phase increased in the hsa_circ_0005397-depleted group in BEL-7404 and HCCLM3 cells (Fig. 4A-B) but decreased in the hsa_circ_0005397-overexpressing group in SK-Hep1 cells (Fig. 4C). The percentage of cells in each phase is shown in Fig. 4D-F. All these results proved that hsa_circ_0005397 promotes growth in HCC cell lines.

### Hsa_circ_0005397 promotes the progression of HCC cell lines through EIF4A3

**Fig. 5:**
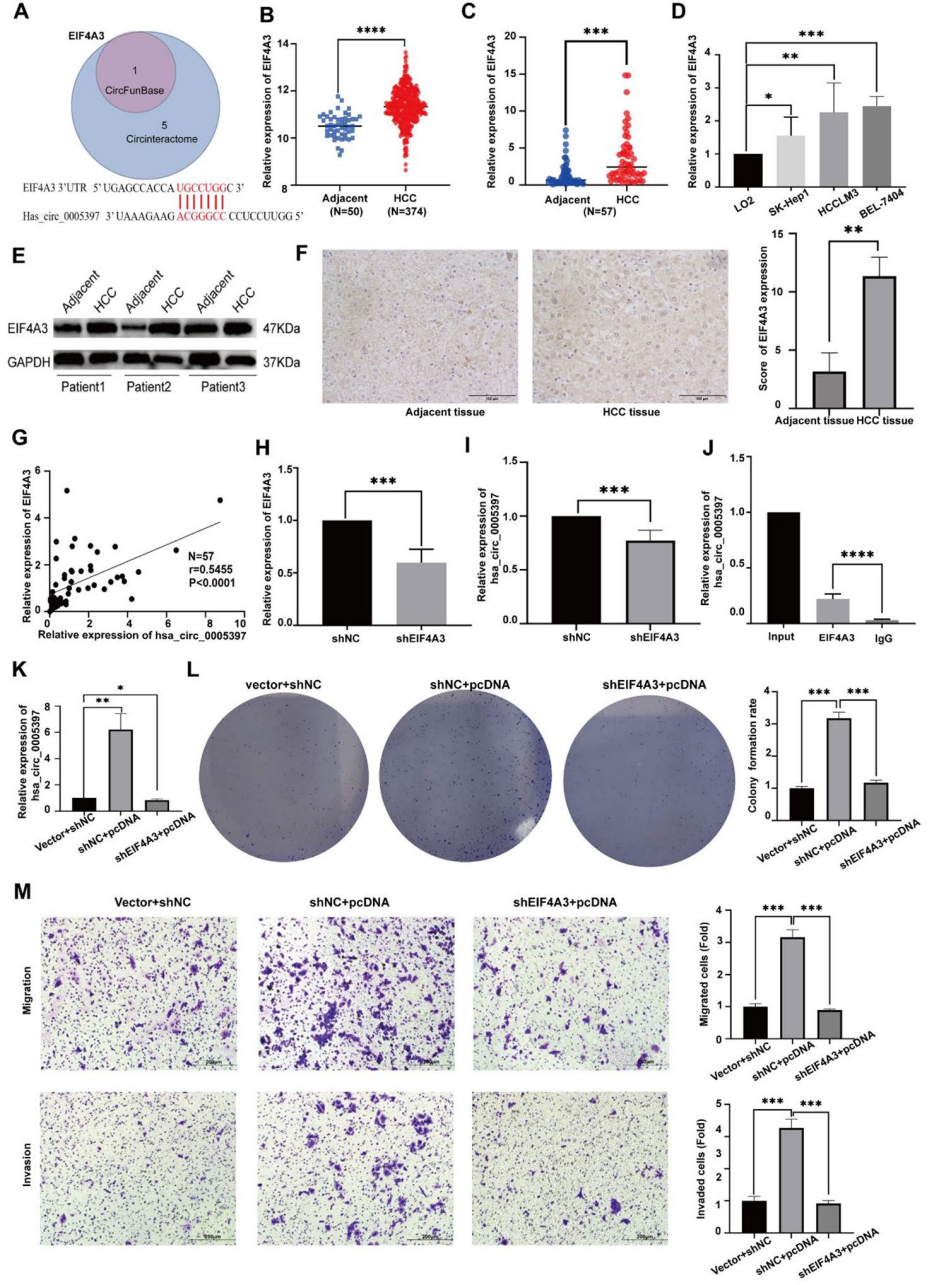
Hsa_circ_0005397 promotes the progression of HCC cell lines through EIF4A3. (**A**) Venn diagram analysis of the two databases (CircInteractome and CircFunBase) identified EIF4A3 as a candidate. (**B**) EIF4A3 expression in HCC tissues from TCGA datasets. (**C**) qRT-PCR detected the expression level of EIF4A3 in HCC tissues(*n* = 57). (**D**) The relative expression level of EIF4A3 in HCC cell lines. (**E**) Western Blot analysis of EIF4A3 in adjacent tissues and paired HCC tissues. (**F**) IHC staining of HCC tissues for EIF4A3 expression. ((magnification, 40x; scale bars, 100 μm). (**G**) Correlation between EIF4A3 and hsa_circ_0005397 mRNA expression in HCC tissues (*n* = 57). (**H**) qRT-PCR detected the expression of EIF4A3 in HCCLM3 cells transfected with shNC or shEIF4A3. (**I**) qRT-PCR analyzed the expression of hsa_circ_0005397 in HCCLM3 cells transfected with shNC or shEIF4A3. (**J**) RIP assay verified that EIF4A3 interacted with hsa_circ_0005397 and IgG was used as a control. (**K**) qRT-PCR detected the expression of hsa_circ_0005397 in HCCLM3 cells transfected with shNC + vector, shEIF4A3 + pcDNA or shEIF4A3 + pcDNA. (**L**) Cell proliferation ability was assessed by colony formation assays after HCCLM3 cells transfected with shNC + vector, shEIF4A3 + pcDNA or shEIF4A3 + pcDNA. Colony counts were counted to analyze colony formation rate, *P < 0.05, **p < 0.01, ***p < 0.001. (**M**) Cell migration and invasion abilities were determined by Transwell assays after HCCLM3 cells transfected with shNC + vector, shEIF4A3 + pcDNA or shEIF4A3 + pcDNA. (magnification, 20x; scale bars, 200 μm), *P < 0.05, **p < 0.01, ***p < 0.001

According to the prediction of Circ Interactome and CircFunBase, EIF4A3 matched to hsa_circ_0005397 (Fig. 5A). We also found that EIF4A3 was overexpressed in HCC tissues from TCGA datasets, paired HCCtissues (*N* = 57) and HCC cell lines (Fig. 5B-D). Moreover, expression of EIF4A3 was also detected by Western Blot and IHC (Fig. 5E-F). What’s more, hsa_circ_0005397 correlated with EIF4A3 significantly positively (Fig. 5G). The efficiency of EIF4A3 depletion in HCCLM3 cells was verified by qRT-PCR (Fig. 5H). After depletion of EIF4A3, expression of hsa_circ_0005397 decreased significantly in HCCLM3 cells (Fig. 5I). We used the RIP assay to verify the relationship between hsa_circ_0005397 and EIF4A3. Compared to the IgG group, hsa_circ_0005397 was enriched in the EIF4A3 group, showing that EIF4A3 could interacted with hsa_circ_0005397 (Fig. 5J). Next, we designed rescue experiments. HCCLM3 cells were co-transfected with pcDNA and shEIF4A3, and qRT-PCR was used to verify expression level of circ_0005397 (Fig. 5K). Moreover, both EIF4A3 inhibition and overexpression hsa_circ_0005397 developed less colonies compared with cells with only hsa_circ_0005397 overexpression (Fig. 5L). The relative migration and invasion cell numbers with both EIF4A3 inhibition and overexpression hsa_circ_0005397 shown less compared with cells with only hsa_circ_0005397 overexpression (Fig. 5M), these results demonstrate that hsa_circ_0005397 may regulate the proliferation of HCC through EIF4A3.

## Discussion

Despite advancements in this field [24, 25], the survival rate of patients with HCC remains unsatisfactory due to challenges in early tumor diagnosis, as well as tumor recurrence and metastasis [26, 27]. Although serum AFP has been widely utilized as a tumor marker for HCC diagnosis and screening, its sensitivity and specificity are not sufficiently high, necessitating further improvements [28, 29]. Recent years, research on circular RNAs (circRNAs) has provided a novel avenue that is expected to contribute to clinical diagnosis and prognosis of HCC [30–32]. Hsa_circ_0005397 was a kind of circRNA, which has been identified to play a key role in several cancers. For instance, hsa_circ_0005397 was upregulated and promoted cell proliferation and invasion in pancreatic cancer [33]. More importantly, hsa_circ_0005397 was overexpressed in HCC tissues, which could promote hepatocellular carcinoma progression by regulating the miR-326/PDK2 axis [21]. In this study, we also verified that hsa_circ_0005397 were markedly overexpressed in both HCC tissues and cell lines. We found that hsa_circ_0005397 had good sensitivity and specificity for diagnosis in HCC patients, and upregulated expression of hsa_circ_0005397 was significantly related to tumor size and stage. To explore the function of has_circ_0005397 in HCC cells, we designed two siRNAs targeting the back splicing junction of has_circ_0005397, the results shown that downregulated expression of has_circ_0005397 may affect cell migration, invasion and proliferation while overexpression of has_circ_0005397 shown the opposite yield, indicating the oncogenic role in HCC growth.

With the development of sequencing and other technologies, the diversity and biological functions of circRNAs are being extensively explored. CircRNAs can sponge miRNAs, bind to RBPs, and regulate gene transcription and translation to regulate tumorigenesis [20, 34, 35]. Unfortunately, previous studies mainly focused on circRNAs serving as miRNA sponges. Actually, circRNAs could also competitively bind to and interact with RBPs [36]. In our study, we found that EIF4A3 was a RBP of hsa_circ_0005397 from bioinformation websites. EIF4A3 plays a crucial role in post-transcriptional regulation and has been reported to interact with RNAs and act as a diagnostic marker in many cancers [37–39]. Recent Studies have demonstrated that EIF4A3 binds to the upstream region to promote circMMP9 expression, showing an important role in mRNA splicing [40]. In addition, EIF4A3 was found to bind to circZFAND6 pre-mRNA transcript upstream region, leading to the high expression of circZFAND6 in breast cancer [41]. All these results concluded that EIF4A3 may be an important circRNA regulator and play important role in the post-transcriptional process. In our study, we found that EIF4A3 was remarkably overexpressed, and the expression of EIF4A3 was positively correlated with the expression of hsa_circ_0005397. Importantly, the expression of hsa_circ_0005397 was downregulated when depletion of EIF4A3, showing that the RBP protein EIF4A3 may affect the transcription level of hsa_circ_0005397. What’s more, the RIP assay was confirmed that EIF4A3 was able to interact with hsa_circ_0005397. Moreover, recent studies have demonstrated that EIF4A3 could regulate EMT process and facilitate tumor progression in HCC [42]. In our study, we found that EIF4A3 inhibition could reverse the overexpression effects of hsa_circ_0005397 on HCC cell proliferation, migration and invasion, showing that hsa_circ_0005397 may regulate HCC progression through EIF4A3. Overall, these findings demonstrated that hsa_circ_0005397 promoted HCC progression and metastasis through EIF4A3. Our study provided evidence for the underlying mechanism of hsa_circ_0005397 functions in the tumorigenesis of HCC, and shed light on the potential biomarkers and therapeutic targets for HCC.

## Conclusion

Our study concluded that overexpression of hsa_circ_0005397 promoted progression and metastasis of HCC through EIF4A3. Regulating the hsa_circ_0005397/EIF4A3 axis would be a potential strategy for HCC.

### Electronic supplementary material

Below is the link to the electronic supplementary material.

Supplementary Material 1

## Data Availability

The datasets used and/or analyzed during the current study available from the corresponding author on reasonable request.
