# Supplementary figures and images for: Hsa_circ_0005397 promotes hepatocellular carcinoma progression through EIF4A3
Source: BMC Cancer. 2024 Feb 21;24:239. doi: 10.1186/s12885-024-11984-6 (PMC10882807; doi:10.1186/s12885-024-11984-6)

**Whole Gel**

**
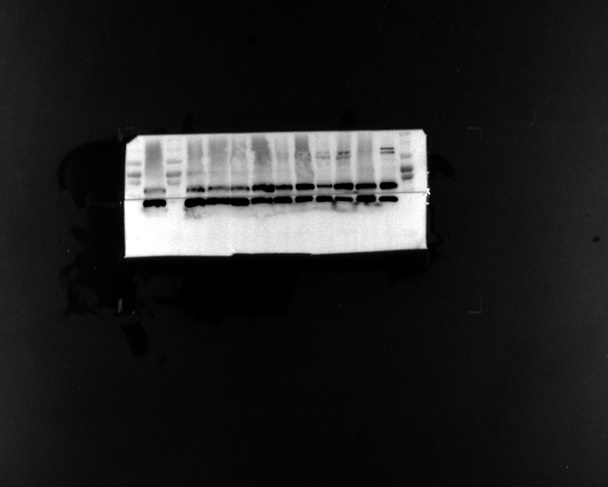
**

**ECL Whole Blots (EIF4A3 47KDa， GAPDH 37KDa）The same gel.**

**
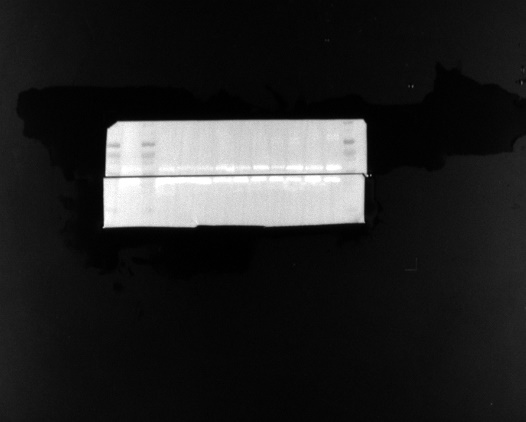
**

**White light (Marker:170 130 100 70 55 40 35 25 15 10KDa)**

Supplement: Supplementary file 1 — Supplementary Material 1 [file 12885_2024_11984_MOESM1_ESM.docx]
